# Copine A Interacts with Actin Filaments and Plays a Role in Chemotaxis and Adhesion

**DOI:** 10.3390/cells8070758

**Published:** 2019-07-21

**Authors:** Matthew J. Buccilli, April N. Ilacqua, Mingxi Han, Andrew A. Banas, Elise M. Wight, Hanqian Mao, Samantha P. Perry, Tasha S. Salter, David R. Loiselle, Timothy A.J. Haystead, Cynthia K. Damer

**Affiliations:** 1 Department of Biology, Central Michigan University, Mount Pleasant, MI 48859, USA; 2 Department of Pharmacology and Cancer, Duke University Medical Center, Durham, NC 27708, USA

**Keywords:** copine, actin, calcium, *Dictyostelium*, cAMP, chemotaxis, adhesion

## Abstract

Copines make up a family of calcium-dependent, phospholipid-binding proteins found in numerous eukaryotic organisms. Copine proteins consist of two C2 domains at the N-terminus followed by an A domain similar to the von Willebrand A domain found in integrins. We are studying copine protein function in the model organism, *Dictyostelium discoideum*, which has six copine genes, *cpnA-cpnF*. Previous research showed that cells lacking the *cpnA* gene exhibited a cytokinesis defect, a contractile vacuole defect, and developmental defects. To provide insight into the role of CpnA in these cellular processes, we used column chromatography and immunoprecipitation to isolate proteins that bind to CpnA. These proteins were identified by mass spectrometry. One of the proteins identified was actin. Purified CpnA was shown to bind to actin filaments in a calcium-dependent manner in vitro. *cpnA^−^* cells exhibited defects in three actin-based processes: chemotaxis, cell polarity, and adhesion. These results suggest that CpnA plays a role in chemotaxis and adhesion and may do so by interacting with actin filaments.

## 1. Introduction

Copines make up a family of calcium-dependent phospholipid-binding proteins found in many eukaryotic organisms [1,2]. Copine proteins contain two C2 domains in the N-terminal half of the protein followed by an A domain similar to the von Willebrand A (VWA) domain in the C-terminal half of the protein [1]. The C2 domain is a calcium-dependent phospholipid-binding motif originally identified in protein kinase C. Most proteins containing a single C2 domain are involved in signaling pathways, while most proteins that have multiple C2 domains are involved in membrane trafficking [3]. The VWA domain is named from the von Willebrand Factor, a plasma and extracellular matrix protein. VWA domains have been studied in integrins and several extracellular matrix proteins, and appear to function as protein-binding domains [4]. Although the exact function of copines is not known, a growing body of evidence suggests that copines may mediate an array of cellular processes by conferring calcium regulation to various signaling pathways [5,6,7,8].

A general hypothesis proposed by Tomsig et al. [9] for how copines may regulate signaling pathways is that specific copines interact with other cellular proteins through their A domains and then either deliver soluble target proteins to membranes or regulate the function of membrane proteins through the action of the C2 domains in response to a rise in intracellular calcium. Tomsig et al. [9] identified more than 20 distinct potential targets of A domains of human copines I, II, and IV using a yeast two-hybrid screen. Among the proteins that were found to associate with human copine A domains were various regulators of phosphorylation, transcription, ubiquitination, cytoskeleton, exocytosis, and mitosis, suggesting that copines carry out many diverse functions.

We are studying the function of copines using the model organism *Dictyostelium discoideum*. We have identified six copine genes in *Dictyostelium discoideum* and have focused our studies on one of the copine proteins, CpnA [10,11,12,13]. Cells lacking the *cpnA* gene (*cpnA^−^*) were previously shown to have defects in cytokinesis, contractile vacuole function, and development [11]. *Dictyostelium discoideum* lives as unicellular haploid amoeba feeding on bacteria. However, when starved, the amoeba will secrete and respond to periodic waves of cAMP to aggregate into a mound. A tip is formed on the mound that elongates into a finger-like structure that falls over to form a slug. The slug is capable of moving toward light and heat in processes called phototaxis and thermotaxis, respectively. When conditions are favorable, slug movement will arrest, and the slug will culminate into a fruiting body consisting of a mass of spores on top of a long thin stalk made up of vacuolated cells [14]. When *cpnA^−^* cells were starved, they were delayed in aggregation to form the mound and then arrested at the slug stage [11]. The slugs formed by *cpnA^−^* cells were bigger than normal slugs, and they were not able to carry out normal phototaxis and thermotaxis [13].

Previous studies in our lab have shown that GFP-tagged CpnA localized to the cytosol in live *Dictyostelium* cells [10,15]. However, when cells were treated with a calcium ionophore in the presence of calcium, GFP-tagged CpnA was found associated with the plasma membrane and intracellular organelles. In addition, in cells primed for aggregation, GFP-tagged CpnA quickly translocated to the plasma membrane, and then back to the cytosol in response to cAMP stimulation, suggesting that CpnA may have a role in cAMP signaling during chemotaxis [15]. To investigate the specific role of CpnA in these processes, we used column chromatography and immunoprecipitation to identify potential binding partners of CpnA. One protein identified by both techniques was actin. Because several of the defects observed in *cpnA^−^* cells are consistent with a defect in the actin cytoskeleton, we explored this interaction further. We found that CpnA binds to actin filaments in a calcium-dependent manner in vitro. Furthermore, cells lacking CpnA exhibited increased adhesion, were defective in their actin polymerization response to cAMP stimulation, and in their ability to sense and move towards a cAMP gradient. 

## 2. Materials and Methods

### 2.1. Dictyostelium Strains and Cell Culture 

The *Dictyostelium discoideum* strain used was NC4A2, an axenic strain derived from the wild-type NC4 strain [16]. NC4A2 cells are referred to as the parental strain hereafter. Cells were grown at 20 °C on plastic culture dishes in HL-5 media (0.75% proteose peptone, 0.75% thiotone E peptone, 0.5% Oxoid yeast extract, 1% glucose, 2.5 mM Na_2_HPO_4_, and 8.8 mM KH_2_PO_4_, pH 6.5) supplemented with penicillin-streptomycin at 60 U/mL. Plasmid transformed cells were cultured in HL-5 media supplemented with 7.5 μg/mL G418. The full-length coding sequence of *cpnA* and the A domain of *cpnA* (bases 1-1000) were amplified by PCR from the cDNA clone, SLI-395 [17]. The PCR fragments were subcloned into the *Dictyostelium* extrachromosomal plasmid, pTX-GFP [18], containing a gene for a variant of green fluorescent protein (GFP, S65A, V68L, and S72A mutations) to produce a fusion protein with a HIS-tag and GFP at the N-terminus of CpnA (GFP-CpnA) and the A domain of CpnA (GFP-Ado). As a control, *Dictyostelium* cells were also transformed with the pTX-GFP plasmid without a *cpnA* cDNA insertion; these cells express a HIS-tagged GFP. The *cpnA* cDNA was also subcloned into the pDXA-GST plasmid [19] to produce a fusion protein with glutathione-S-transferase (GST) at the N-terminus and a HIS-tag at the C-terminus of CpnA. *Dictyostelium* cells were transformed with plasmids by electroporation. 

Previously, a *cpnA* knockout (KO) strain (*cpnA^−^*) was created by replacing the *cpnA* gene with the blasticidin S resistance gene (*bsr*) in the axenic strain, NC4A2 [11]. The *cpnA* knockout DNA construct included PCR fragments of approximately 1 kb upstream (5’) and downstream (3’) of the *cpnA* gene that were ligated into the pBSIIbsr plasmid to flank the *bsr* gene. Another *cpnA* knockout strain (*cpnA^−cre^*) was made using the pLPBLP plasmid, which contains the *bsr* cassette bookended by loxP sites [20]. The 5’ and 3’ flanking regions of the *cpnA* gene were removed from the pBSIIbsr plasmid, and ligated into the pLPBLP plasmid at the KpnI and HindIII, and BamHI and NotI restrictions sites, respectively. The plasmid DNA was linearized and electroporated into NC4A2 cells. Clonal populations were selected by resistance to blasticidin (10 µg/mL) and screened for expression of CpnA by western blot with rabbit polyclonal antisera raised against a bacterially expressed protein fragment of CpnA. Cell lines that did not express CpnA were also screened by PCR using primers designed to amplify the middle of the *cpnA* gene. A digoxigenin labeling and detection kit (Roche Diagnostics, Indianapolis, IN, USA) was used in a Southern blot to verify the *bsr* gene was found once in the genome and had replaced the *cpnA* gene in the genome (Figure S1).

### 2.2. Isolation of CpnA Binding Proteins using Column Chromatography

*Dictyostelium* NC4A2 cells expressing GFP-CpnA were grown to 2 × 10^6^ cells/mL in 2 L of HL-5 media in a shaking incubator at 20 °C. Cells were centrifuged at 7000 RPM in a GSA Beckman rotor. Pellets were resuspended in 5 mL of cold homogenization buffer (50 mM HEPES, pH 7.4, 150 mM NaCl, with protease inhibitors). Cells were disrupted with two passes through a French Press. After adding 5 mM EGTA, the cell lysate was centrifuged at 27,000× *g* for 15 min at 4 °C. The supernatant containing the GFP-CpnA protein was removed. Liposomes were made by dissolving 200 mg of bovine brain lipids (cat#B1502, Sigma-Aldrich, St. Louis, MO, USA) in 20 mL of homogenization buffer. The lipids were sonicated, then pelleted at 50,000 RPM for one hour in an ultracentrifuge. The liposomes were resuspended in the cell lysate supernatant containing the GFP-CpnA protein, and CaCl_2_ was added to a final concentration of 8 mM CaCl_2_. The liposomes were centrifuged again at 50,000 RPM for one hour. The supernatant was discarded, and the pelleted liposomes were washed in Wash Buffer (25 mM HEPES, pH 7.4, 2 mM CaCl_2_) twice. After the last spin, the liposome pellet was resuspended in Extracting Buffer (25 mM HEPES, pH 7.4, 10 mM EGTA) and the liposomes were pelleted again. The supernatant containing the GFP-CpnA was removed, and the lipids were pelleted and resuspended in Extracting Buffer two more times. The supernatants containing the GFP-CpnA were run over a PD-10 Sephadex G-25M desalting column (Amersham Biosciences, Piscataway, NJ, USA) to remove the EGTA. The desalted protein mixture was then loaded onto a 1 mL HisTrap FF column (GE Healthcare, Waukesha, WI, USA), equilibrated with binding buffer (20 mM sodium phosphate, 500 mM NaCl, 45 mM imidazole, pH 7.4). The column was washed with binding buffer and proteins were eluted with elution buffer (20 mM sodium phosphate, 500 mM NaCl, 500 mM imidazole, pH 7.4). The eluted protein mixture was desalted using a PD-10 desalting column and analyzed by SDS-PAGE. 

The resulting purified GFP-CpnA was coupled to agarose beads using an Aminolink Plus Immobilization kit according to the manufacturer’s instructions (cat#44894, ThermoFisher Scientific, Waltham, MA, USA). *Dictyostelium* NC4A2 cells were grown in 1 L of HL-5 media in a shaking incubator at 20 °C. Cells were centrifuged at 7000 RPM in a GSA Beckman rotor. Pellets were resuspended in 5 mL of cold homogenization buffer. EGTA was added to a final concentration of 2 mM and cells were disrupted with two passes through a French Press. The cell lysate was centrifuged at 50,000 RPM in an ultracentrifuge for 45 min at 4 °C. CaCl_2_ was added to the supernatant to a final concentration of 10 mM, and the pH was adjusted to 7.4. The supernatant containing soluble proteins was applied to the GFP-CpnA-linked agarose column and a control column containing agarose beads. Both columns were washed twice in homogenization buffer containing 2 mM CaCl_2_. Proteins were eluted from the columns with a low pH buffer (0.2 M glycine, pH 2.5) and these column fractions were neutralized with 1 M Tris, pH 9. Column fractions were analyzed by SDS-PAGE and stained with Coomassie Blue. 

### 2.3. Isolation of CpnA Binding Proteins using Immunoprecipitation

*Dictyostelium* NC4A2 cells (2 × 10^8^) expressing GFP, GFP-Ado, or GFP-CpnA were harvested from plates and pelleted by centrifugation at 1500 RPM for 5 min. Cells were washed twice with 5 mL of HEPES Buffer (50 mM, pH 7.4) at 4 °C. Cells were then resuspended in 2 mL of Lysis Buffer (50 mM HEPES, 100 mM NaCl, 2 mM CaCl_2_, 10% Sucrose, 0.3% NP-40, pH 7.4 with protease inhibitors). Cell lysates were centrifuged at 14,000 RPM for 15 min. Supernatants (2 mL) and 0.5 mL HEPES buffer were applied to HEPES buffer equilibrated PD-10 desalting columns, then 3.5 mL of HEPES buffer was applied to each column, and the flow-through was collected. Protein A agarose beads (Roche Diagnostics) were washed four times with 4 mL of HEPES Buffer and then resuspended in HEPES Buffer to create a 50% *v*/*v* bead slurry. Cell supernatants were pre-cleared twice by adding 50 µL of 50% *v*/*v* protein A agarose beads, incubating at 4°C with rocking for 1 hour, and then centrifuging at 3000× *g* for 10 min. The pre-cleared supernatant was then incubated with 3 µL of anti-GFP antibody (ab-290, Abcam, Cambridge, UK) for 1 h with rocking, then incubated with protein A agarose beads for an additional hour. The beads were pelleted by centrifugation at 4500 RPM for 30–45 sec and washed four times with 1 mL of HEPES Buffer. After the last wash, the supernatant was discarded. The pelleted beads were incubated with 50 µL of sample buffer at 95 °C for 5 min. The beads were pelleted again with centrifugation at 10,000 × g for 5 min. Supernatant samples were analyzed by SDS-PAGE and stained with Coomassie Blue. 

### 2.4. Identification of Proteins using Mass Spectrometry

Proteins were analyzed by SDS-PAGE and stained with Coomassie Blue. Visible bands were excised from the gel manually and cut into small pieces approximately 1 mm × 1 mm. These gel pieces were washed three times with water, then washed twice with 1:1 acetonitrile: 100 mM ammonium bicarbonate. To further prepare the gel pieces for digestion, the gel pieces were then dehydrated in 100% acetonitrile. After removing all acetonitrile, 25 µL of porcine trypsin (Promega, Madison, WI, USA) dissolved in 25 mM ammonium bicarbonate at a concentration of 4 µg/mL was added to the gel pieces. The gel pieces were then kept at room temperature (RT) overnight (approximately 12–16 h). Following digestion, the supernatant was transferred to a second tube, and acetonitrile was added to the gel pieces to complete the extraction of digested peptides. This extract was added to the first supernatant, and this combined solution, containing the extracted peptides was frozen and lyophilized. The peptides were resuspended in 5 µL of 100:99:1 acetonitrile: water: trifluoroacetic acid immediately prior to spotting on the MALDI target. 

For matrix-assisted laser desorption/ionization (MALDI) analysis, the matrix solution consisted of alpha-cyano-4-hydroxycinnamic acid (Aldrich Chemical Co. Milwaukee, WI, USA) saturating a solution of 1:1:0.01 acetonitrile: 25 mM ammonium citrate: trifluoroacetic acid. Approximately 0.15 µL of peptide solution was spotted on the MALDI target immediately followed by 0.15 µL of the matrix solution. This combined solution was allowed to dry at RT. MALDI MS and MS/MS data were then acquired using the ABSCIEX 5800 TOFTOF Mass Spectrometer. Resultant peptide mass fingerprint and peptide sequence data were submitted to the UniProt database using the Mascot search engine to which relevance is calculated, and scores are displayed (Tables S1 and S2).

### 2.5. Glutathione Agarose Chromatography

*Dictyostelium* NC4A2 cells expressing GST-tagged CpnA were harvested from plates and counted with a hemocytometer. The cells were pelleted at 1500 RPM for 5 min at 4 °C, and the supernatant was discarded. Lysis buffer containing 5 mM EGTA, 1% Triton X-100, 1 mM DTT and 1× protease inhibitor in PBS (137 mM NaCl, 2.7 mM KCl, 4.3 mM Na_2_HPO_4_, 1.47 mM KH_2_PO_4_, pH 7.4) was used to resuspend the pelleted cells, and the cell suspension was incubated on ice for 30 min. Glutathione agarose beads (Sigma, 50 mg) were suspended in 5 mL of deionized water, placed on the rotator, and incubated at RT for 30 min. The beads were washed three times in PBS by pelleting at 3000 RPM for 5 min at 4 °C. The beads were resuspended in 150 µL of PBS to make a 50% bead slurry. The cells in lysis buffer were spun in a microcentrifuge at 14,000 RPM for 5 min at 4 °C. The bead slurry was added to the cell lysate supernatant and placed on the rotator at RT for 30 min. The beads were washed by pelleting at 3000 RPM for 5 min at 4 °C four times in PBS, and two times using lysis buffer. The beads were washed with 5 mL of 10 mM Tris (pH 8.0), then incubated for 10 min on a rotator at RT in elution buffer (10 mM reduced glutathione in 50 mM Tris-HCl, pH 8.0). The beads were washed three times with elution buffer, and the supernatants were analyzed by SDS-PAGE (Figure S2) and stored at −20 °C. The concentration of the purified GST-CpnA was determined using a BIO-RAD protein assay kit.

### 2.6. F-actin Binding Assay

Purified GST-CpnA samples were thawed on ice and spun at 150,000× g (50,000 RPM) for 1 h at 4 °C. The supernatant was removed and kept on ice. An F-actin stock was prepared as described in the published protocol (Cytoskeleton, Inc.). Three samples were prepared for the F-actin binding assay: (1) An F-actin control sample made by mixing 40 µL F-actin stock (1 mg/mL) and 10 µL of glutathione elution buffer; (2) a no actin protein control made by mixing 10 μL GST-CpnA protein (20 μg/mL) and 40 μL F-actin buffer; and (3) an experimental sample made by mixing 40 μL F-actin stock (1 mg/mL) and 10 μL GST-CpnA protein (24 μg/mL). In addition, the same three samples were prepared again, but 2 mM EGTA was added to the samples. All samples were incubated at RT for 30 min then spun at 150,000× g in an Airfuge for 1.5 h at 24 °C. The supernatants were removed, and 10 μL 6× sample buffer was added into each supernatant. The pellets were resuspended with 30 µL 2× sample buffer. All the samples were analyzed by SDS-PAGE and stained with Coomassie Blue. A western Blot was performed for the supernatants and pellets from the samples that included GST-CpnA using an HRP conjugated anti-GST antibody (1:5000). An ECL chemiluminescence kit (Amersham Biosciences) and the Kodak Gel Logic 2200 were used to image the blot.

### 2.7. Dynabead Immunoprecipitations

*Dictyostelium* NC4A2 cells (5 × 10^7^) expressing GFP, GFP-Ado, and GFP-CpnA were harvested from plates in HL-5 media. Cells were then centrifuged at 1500 RPM for 5 min, the supernatant was removed, and the cells were washed with 5 mL of 50 mM HEPES buffer, pH 7.4. The cells were centrifuged again, the supernatant was removed, and the cells were resuspended in 1.5 mL of Lysis buffer (50 mM HEPES, pH 7.4, 100 mM NaCl, 2mM EGTA, 10% *w*/*v* sucrose, and 0.3% NP-40). Cell lysates were centrifuged in a microcentrifuge at 14,000 RPM for 10 min. Magnetic Protein G Dynabeads (Life Technologies, Carlsbad, CA, USA) were washed in PBS and incubated with 2 μL of rabbit polyclonal anti-GFP antibody (Abcam, ab290) for 10 min. The beads were resuspended with the cell lysate and incubated for 30 min. The beads were washed three times with 200 μL of 50 mM HEPES, pH 7.4. After the final wash, the beads were resuspended with 100 μL 50 mM HEPES, pH 7.4 and transferred to new tubes.

G-actin and F-actin stocks were prepared as described in the published protocol (Cytoskeleton, Inc., Denver, CO, USA). The F-actin or G-actin stock (40 μL) was added to the beads-antibody-protein complex and incubated for 10 min at RT; then the beads were washed once with 200 μL of 50 mM HEPES buffer. The same protocol was followed again, but EGTA (2mM) was added to the actin stocks. The beads were precipitated out of solution using the magnet and then resuspended in 40 μL of 2× sample buffer and heated at 95 °C for 5 min. Samples were analyzed by western blotting with a mouse monoclonal anti-actin antibody (cat# sc-47778, Santa Cruz Biotechnology, Inc) diluted 1:2000 and anti-mouse HRP-conjugated antibody diluted 1:15,000. An ECL chemiluminescence kit (Amersham Biosciences, ) and the Kodak Gel Logic 2200 was used to image the blot. Blots were re-probed with a mouse monoclonal anti-GFP antibody (cat# sc-9996, Santa Cruz Biotechnology, Inc., Santa Cruz, CA, USA) diluted 1:2000 and the same secondary antibody diluted 1:15,000.

### 2.8. Folate and cAMP Chemotaxis Assays

*Dictyostelium* NC4A2 and *cpnA* KO cells were grown in HL-5 media in a shaking incubator at 20 °C at 180 RPM. Small wells were made in agar plates containing Development Buffer ((DB) 5 mM Na_2_HPO_4_, 5 mM KH_2_PO_4_). DB alone or with 100 µM folate or 100 µM cAMP was placed in the wells. For folate chemotaxis assays, cells were washed once in DB and then resuspended in DB at a concentration of 2.5 × 10^8^ cells/mL. Cells were placed as 1 µL drops 5 mm away from each well. For cAMP chemotaxis assays, cells were washed in DB and resuspended at 1 × 10^7^ cells/mL. Cells were placed in a shaking incubator at 180 RPM for eight hours at 20 °C. Starved cells were pelleted, washed once in DB, and then resuspended in DB containing 2 mM caffeine so that the final cell concentration was 2.5 × 10^8^ cells/mL. The cells were placed as 1 µL drops 5 mm from each well. The plates were placed in a humidity chamber for 3 h at 20 °C. Cell drops were imaged using a Leica dissecting microscope at 12.5× magnification. The distance moved by cells, away from the edge of the cell drop, was measured in the direction towards the chemoattractant well, as well as in the opposite direction, using ImageJ. Two trials (three replicates per trial) for each cell type and chemoattractant were performed. Distance data towards and away from chemoattractant were analyzed separately by a 2-way ANOVA with post-hoc comparisons using the Tukey method. 

### 2.9. cAMP Gradient Chemotaxis Assay

*Dictyostelium* NC4A2 and *cpnA* KO cells were harvested from plates, counted with a hemocytometer, centrifuged at 1,500 RPM for 5 min at 4°C, washed twice with KK2 buffer (16.2 mM KH_2_PO_4_, 4.0 mM K_2_HPO_4_), and resuspended in KK2 buffer at 1 × 10^7^ cells/mL. The cells were starved for 1 hour in shaking suspension at 150 RPM at 20 °C, and subsequently pulsed with 50 nM cAMP every 6 min for 5 h with shaking at 150 RPM. Cells were diluted to 7.5 × 10^5^ cells/mL and treated with 2.5 mM caffeine for 15 min, while shaking at 150 RPM. Cells were placed on coverslips for 10 min and placed on the Dunn Chemotaxis Chamber (DCC100, Hawksley, Sussex, UK) and sealed with a hot wax mixture (Vaseline, paraffin, beeswax, 1:1:1). For control experiments with no cAMP gradient, the inner well was filled with KK2 buffer solution with 2.5 mM caffeine. For cAMP gradient experiments, the inner well was filled with KK2 buffer solution with 2.5 mM caffeine and 5 µM cAMP. Time-lapse images of cells were captured with a Leica DFC7000 T camera and a Leica DMi8 light microscope using a 20× DIC objective every 10 s for 10 min. Trials for each cell line with and without a cAMP gradient were performed at least twice for each cell line on different days, except for the pulsed *cpnA^−^* cells in which there was only one trial with and without a cAMP gradient. Time-lapse images were taken from 2–3 different areas of the Dunn chamber within the same trial. The ImageJ manual tracking plug-in was used to track the movement of all cells (~15–20 cells) that appeared viable and stayed within the field of view. To measure directionality, the changes in x and y coordinates every 10 s for 10 min were averaged for each cell. The change in x and y averages from each cell were averaged for each cell type and analyzed for differences among the cell types using *t*-tests. Individual cells from all trials, which included 2–6 movies, were analyzed for velocity and roundness using ImageJ. An average and standard error were calculated from the averages of velocity and roundness for each movie and these averages were analyzed by a 2-way ANOVA with post-hoc comparisons using the Tukey method. 

### 2.10. Actin Polymerization in Response to cAMP Stimulation Assay

*Dictyostelium* NC4A2 and *cpnA* KO cells were harvested from plates, counted with a hemocytometer, centrifuged at 1500 RPM for 5 min at 4 °C, washed twice with KK2 buffer, and resuspended in KK2 at 1 × 10^7^ cells/mL. The cells were starved for 1 h in shaking suspension at 150 RPM at 20 °C, and subsequently pulsed with 50 nM cAMP every 6 min for 5 h with shaking at 150 RPM. Cells were treated with 2.5 mM caffeine for 15 min, while shaking at 150 RPM. Cells (250 µL) were removed from the flask for the 0-s timepoint. Cells were treated with 100 µM cAMP and 100 mM dithiothreitol (DTT), and 250 µL of cells were removed from the flask at 5, 10, 15, 20, 40, and 60 second timepoints and subsequently treated with 250 µL of 2× lysis buffer (1% Triton X-100, 5 mM EGTA, 2 mM MgCl_2_, 4 mM ATP, 20 mM MES, pH 6.8) on ice. Samples were centrifuged at 13,000 RPM for 2 min at 4 °C. The supernatants were removed, placed in 1 mL of acetone, and stored in -20 °C overnight. Pellets were resuspended in 35 µL of 2× SDS sample buffer and boiled at 95 °C for 5 min. Supernatants were removed from −20 °C, samples were centrifuged at 13,000 RPM for 15 min. The supernatants were discarded, and the pellets were air dried to remove any excess acetone and then resuspended in 35 µL of 2× SDS sample buffer and boiled at 95 °C for 3 min. Pellet and supernatant protein samples were separated by SDS-PAGE using BIO-RAD TGX Stain-Free gels. Gels were imaged with a BIO-RAD ChemiDoc Touch imaging system. Densitometry analysis was performed using ImageJ to measure background-subtracted band intensities. Percent F-actin was calculated by dividing the actin band intensity of the pellet by the sum of the pellet and supernatant actin bands at each timepoint. The data were normalized to the 0-timepoint. These experiments were carried out three times from the same pulsed cell populations for each cell type. The normalized data at each timepoint from the three experiments were averaged, and then those means were averaged from two different pulsed cell populations of *cpnA^−^* and *cpnA^−cre^* cells and three different pulsed cell populations of parental NC4A2 cells. Differences between the averages for each cell type at timepoints 0 and 5 s were analyzed by *t*-tests. 

### 2.11. Adhesion Assay

*Dictyostelium* NC4A2 and *cpnA* KO cells were harvested from plates and pelleted by centrifugation at 1500 RPM for 5 min. Cells were resuspended in HL-5 at 1 × 10^6^ cells/mL, and 2 mL of cells were allowed to settle on 35 × 10 mm Petri dishes. Cells that had not adhered to the surface of the Petri dish after 20 min were removed by replacing the media with fresh HL-5. Adherent cells were imaged with a Nikon TE2000 microscope with a 20× phase-contrast objective in 5 marked spots on the Petri dishes, one in the middle and four around the periphery of the dish. Plates were placed on a rotator for 15 min at 50, 75, or 100 RPM. Cells that had separated from the bottom surface of the Petri dish while shaking were removed by replacing the media with fresh HL-5. Images were taken at the same marked spots on the Petri dishes after rotation. The number of cells in each image were then counted using the Cell Counter plugin in ImageJ and averaged for each RPM for three trials. The percent difference of cells still adherent after shaking was calculated by subtracting the average number of cells remaining from the average number of cells present before shaking and divided by the number of cells present before shaking. The percent difference was averaged from three trials. Differences between the cell types at each RPM were analyzed by *t*-tests. 

## 3. Results

### 3.1. Actin Identified as a Potential Binding Partner of CpnA by Column Chromatography and Immunoprecipitation

**Figure 1:**
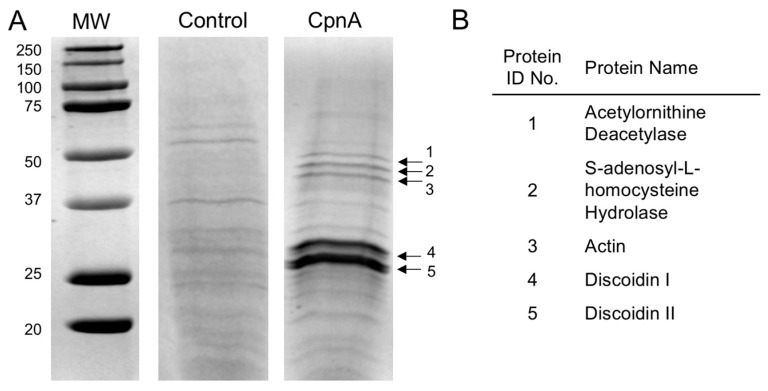
Identification of potential CpnA binding partners by column chromatography. (**A**) Eluates from a control column (Control) and a CpnA-linked column (CpnA) lysate were analyzed by SDS-PAGE. Protein standards (MW) are in kDa. (**B**) Protein bands (1–5) eluted from the CpnA-linked column were identified by mass spectrometry.

We first used column chromatography to identify proteins that interact with CpnA. A HIS-GFP-tagged full-length version of CpnA was overexpressed in *Dictyostelium* cells and purified. The purified protein was covalently linked to agarose beads, and the beads were placed in a column. *Dictyostelium* cell lysates were run over the CpnA-linked agarose bead column and a control column with beads only. Bound proteins were eluted with a low pH buffer. Peak fractions eluted from the control column and the CpnA column were analyzed by SDS-PAGE (Figure 1). Some of the prominent protein bands that were found in the CpnA column eluate were identified by mass spectrometry (Figure 1, Table S1). Five different proteins were identified: Acetylornithine deacetylase, S-adenosyl-L-homocysteine hydrolase, actin, and discoidins I and II. 

**Figure 2:**
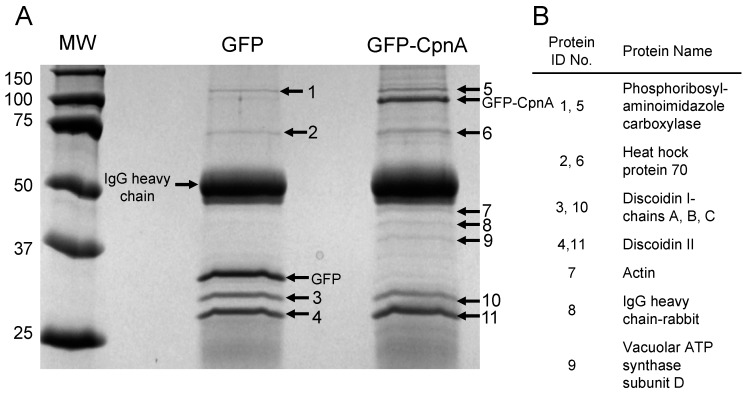
Identification of potential CpnA binding partners by immunoprecipitation. (**A**) Immunoprecipitations performed with an antibody to GFP using cells expressing GFP and GFP-CpnA were analyzed by SDS-PAGE. Protein standards (MW) are in kDa. (**B**) Protein bands (1–11) were identified by mass spectrometry.

We also used another technique to identify potential binding partners of CpnA. *Dictyostelium* cells expressing GFP-CpnA or GFP were used in immunoprecipitations (IPs) with a polyclonal antibody to GFP. IPs were analyzed by SDS-PAGE (Figure 2). Some of the prominent protein bands in both IPs were identified by mass spectrometry (Figure 2, Table S2). Several of the proteins were found in both the GFP control and GFP-CpnA IPs, including discoidins I and II. Three protein bands (Figure 2A, bands 7, 8, 9) appeared to be enriched in the GFP-CpnA eluate compared to the GFP eluate. One of these bands was identified as actin (Figure 2, band 7). Because actin was identified by both techniques and previous research indicated that cells lacking *cpnA* exhibited phenotypes consistent with a defect in the actin cytoskeleton [11], we decided to explore the interaction between CpnA and actin further. 

### 3.2. CpnA Binds to F-Actin, but Not G-actin, in a Calcium-Dependent Manner

**Figure 3:**
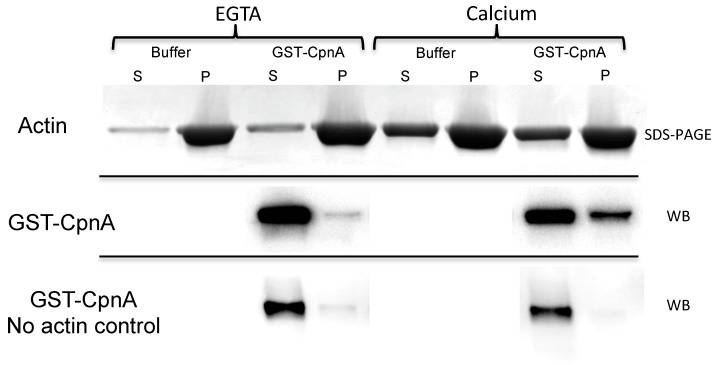
CpnA binds actin filaments in a calcium-dependent manner. Purified GST-CpnA was incubated with F-actin in the presence and absence of calcium. F-actin was pelleted in an ultracentrifuge, and both the supernatant (S) and pellet (P) were analyzed by SDS-PAGE (Actin) and western blot (GST-CpnA) with an anti-GST antibody. GST-CpnA was also centrifuged in the absence of F-actin (No actin control) and both the supernatant (S) and pellet (P) were analyzed by western blot with an anti-GST antibody. The F-actin binding experiments were performed at least three times, and representative blots are shown.

To determine if CpnA binds directly to actin, we performed F-actin binding assays with purified actin and purified GST-tagged CpnA. The GST-tagged CpnA was expressed and purified from *Dictyostelium* cells (Figure S2). The purified GST-CpnA was incubated with F-actin, made from actin purified from human platelets, in the presence and absence of calcium. The F-actin was pelleted by centrifugation. The supernatants containing non-polymerized G-actin and pellets containing the polymerized F-actin were analyzed by SDS-PAGE and Coomassie Blue stain (Figure 3, top panel). GST-CpnA was detected in the same samples with a western blot using an antibody to GST (Figure 3, middle panel). We found that some of the CpnA pelleted with the F-actin, but only in the presence of calcium. Because copines have been shown to oligomerize on membrane surfaces [21], we also performed a control experiment in which purified GST-CpnA was spun down in the ultracentrifuge without F-actin, in the presence and absence of calcium. The GST-CpnA was found only in the supernatant and, therefore, did not pellet on its own in the presence of calcium (Figure 3, bottom panel). These results indicate that GST-CpnA interacts with F-actin in a calcium-dependent manner.

**Figure 4:**
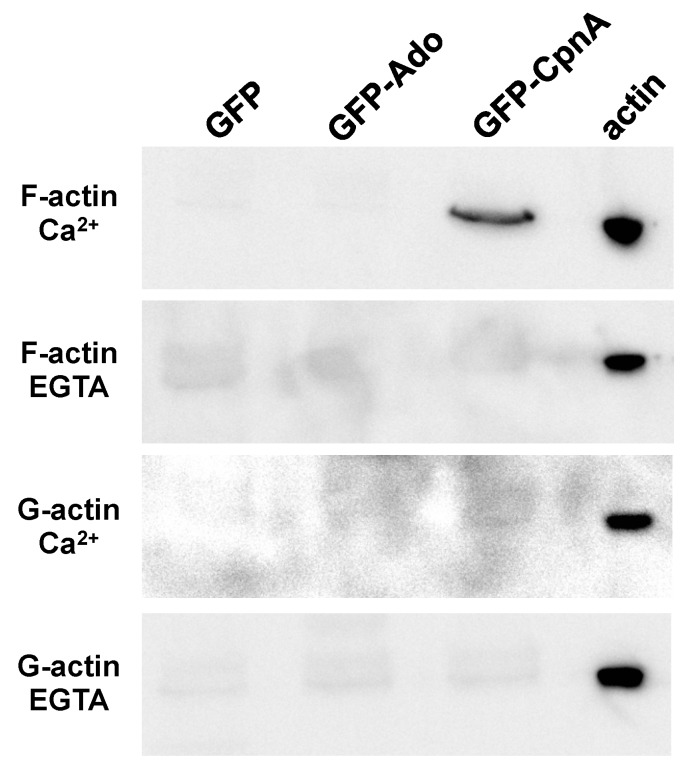
CpnA binds F-actin, but not G-actin. Immunoprecipitations with an antibody to GFP were performed with cells expressing GFP, GFP-Ado, and GFP-CpnA. Precipitated proteins were incubated with F-actin or G-actin and precipitated again. IPs were analyzed using a western blot with an antibody to actin. Actin alone (actin) was also run on the gel as a positive control. The experiment with each cell type was performed at least three times, and representative blots are shown.

To determine if CpnA is able to bind to G-actin, immunoprecipitations using an antibody to GFP were carried out with cells expressing either GFP, GFP-CpnA, or GFP-Ado, which contains the A domain and lacks the C2 domains of CpnA. However, this time, we incubated the immunoprecipitations with F-actin or G-actin, in the presence or absence of calcium. We then collected the beads again, washed the beads once, and analyzed the immunoprecipitations by western blot with an antibody to actin. We found that F-actin co-precipitated with GFP-CpnA, but not GFP, in the presence of calcium (Figure 4). When the immunoprecipitations were performed in the absence of calcium, F-actin did not co-precipitate with any of the immunoprecipitated proteins (Figure 4). When the same immunoprecipitations were performed again and incubated with G-actin, G-actin did not co-precipitate with any of the GFP proteins in the presence or absence of calcium (Figure 4). In a few of the trials, we observed F-actin, but not G-actin, to co-precipitate with GFP-Ado (Figure S3). This suggests that CpnA interacts with actin through its A domain. To verify that GFP, GFP-Ado, and GFP-CpnA were immunoprecipitated in each assay, we also probed the same blots with an antibody to GFP (Figure S4). Overall, these experiments indicate that CpnA binds to F-actin, but not G-actin, in a calcium-dependent manner. 

### 3.3. Creation of a cpnA^−cre^ Mutant Cell Line

Previously we created a *cpnA* knockout cell line (*cpnA^−^*) using homologous recombination to replace the *cpnA* gene with the blasticidin resistant gene (*bsr*) in the *Dictyostelium* NC4A2 axenic strain [11]. Using the same *Dictyostelium* parental strain, NC4A2, we created another cell line lacking the *cpnA* gene. This newly made cell line (*cpnA^−cre^*) was created by homologous recombination, again replacing the *cpnA* gene with the *bsr* gene; however, *loxP* sites flanked the *bsr* gene. The *loxP* sites can be used to remove the *bsr* gene from the genome so that we can use the *bsr* gene again to replace a different copine gene and make multiple copine gene KOs in the same cell line. NC4A2 cells were transformed with the cre-lox plasmid containing the *cpnA* gene flanking sequences. Transformed cells were selected by blasticidin and then screened by western blot using an antibody to CpnA. Cells lacking CpnA expression were further analyzed by PCR and Southern blot to verify the *cpnA* gene was replaced with the *bsr* gene and that the *bsr* gene was found only once in the genome (Figure S1). 

Expression of GFP-CpnA in *cpnA^−^* cells does not rescue all phenotypic defects [11], so it is important to show that several independent *cpnA* KO clones have the same phenotype. Therefore, we tested both *cpnA* KO cell lines in all of the assays used to explore CpnA’s role in three actin-based processes: chemotaxis, polarity, and adhesion. 

### 3.4. cpnA KO Cells Are Defective in Folate and cAMP Chemotaxis

**Figure 5:**
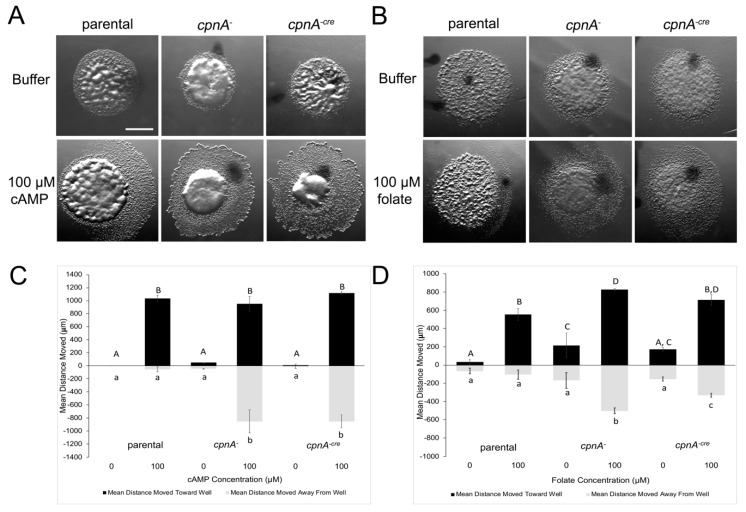
*cpnA* KO cells exhibit chemotaxis defects. cAMP and folate chemotaxis assays were performed with parental, *cpnA^−^*, and *cpnA^−cre^* cells. Cells were plated on agar plates 5 mm away from wells containing either buffer or 100 μM cAMP (A) or 100 μM folate (**B**). Images (**A**,**B**) were taken 3 h later. Wells were directly to the right of each cell drop in each image. Scale bar =1mm. (**C**) Distances cells moved towards and away from wells with cAMP. (**D**) Distances cells moved towards and away from wells with folate. Three replicates within the same trial for each cell type and chemoattractant were averaged, and then the means from 2–3 trials were averaged. Error bars = standard error. Replicate data were analyzed by a 2-way ANOVA, with post-hoc comparisons using the Tukey method, *p* < 0.05. Means with different letters are significantly different.

In the absence of nutrients, unicellular amoeba will secrete and respond to cAMP. Cells will secrete cAMP approximately every six minutes, which in turn results in the cells aligning head-to-tail and streaming into mounds creating aggregation centers [22]. Previously, we showed that starved *cpnA^−^* cells were delayed in aggregation [11], suggesting that they have a defect in cAMP chemotaxis. To further investigate the possible role of CpnA in chemotaxis, we compared the parental strain, NC4A2, and the two *cpnA* KO strains (*cpnA^−^*, *cpnA^−cre^*) in cAMP and folate chemotaxis assays. Vegetative cells will move towards folate, while starved cells that have begun the developmental cycle will express cAMP receptors and move towards cAMP. Therefore, we used vegetative cells for the folate assays and cells that had been starved for 8 h for the cAMP assays. Cells were placed as small dots on agar plates ~5 mm away from wells containing 100 µM concentrations of folate or cAMP. Images of the cells were taken 3 h after plating and the distance cells moved away from the original dot was measured in two directions: (1) toward the well; and (2) in the opposite direction away from the well. Representative images taken three hours after plating of all three cell types are shown in Figure 5A,B. The well-containing either buffer or chemoattractant was to the direct right of each cell dot shown in the image. 

For the cAMP assays (Figure 5A,C), all three cell types moved significantly farther when placed near a well containing cAMP versus a well containing buffer. The parental cells fanned out from the original cell dot towards the cAMP containing well with little movement in the opposite direction away from the well. In contrast, both *cpnA* KO cells moved out from the original dot in all directions (Figure 5A). There was no significant difference in the distance moved in the direction towards the well-containing cAMP for all three cell strains; however, the *cpnA* KO cells moved significantly farther in the opposite direction away from the well than the parental strain (Figure 5C). This suggests the *cpnA* KO cells do not have a defect in movement or detection of the cAMP, but a defect in directional movement towards the cAMP.

For the folate assays (Figure 5B,D), the *cpnA* KO cells moved a little farther than the parental cells regardless if they were placed next to the folate wells or buffer wells. *cpnA* KO cells exhibited the same behavior as in the cAMP assays in that the *cpnA* KO cells moved significantly farther than the parental cells in the opposite direction of the folate containing well (Figure 5B,D). The KO phenotype appeared to be less severe in the folate assays than in the cAMP assays in that the *cpnA* KO cells moved farther in the direction toward the well containing folate than in the direction away from the well. Again, these results suggest that *cpnA* KO cells move in response to folate, but have a defect in the directional movement, given that the cells moved in the opposite direction of the folate. 

### 3.5. cpnA KO Cells Fail to Polarize and Are Unable to Move Directionally within a cAMP Gradient

**Figure 6:**
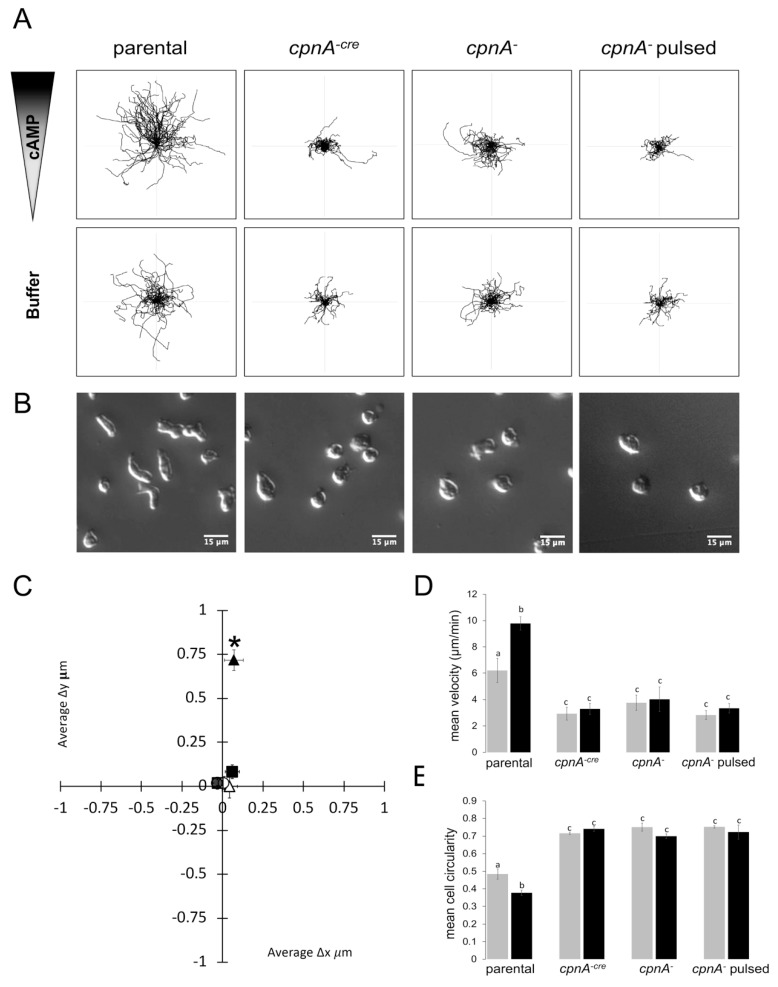
*cpnA* KO cells exhibit defects in directional sensing, motility, and polarity. Parental, *cpnA*, and *cpnA^−cre^* cells were starved and placed in a Dunn chamber within a cAMP gradient (cAMP) or no gradient (buffer). *cpnA^−^* cells were also pulsed with cAMP every six minutes for six hours (*cpnA^−^* pulsed) before being placed in the Dunn chamber. Images were taken every 10 s for 10 min using DIC microscopy and individual cells were tracked using ImageJ. (**A**) Representative cell trajectories from one trial. (**B**) Representative DIC images of cells within a cAMP gradient. (**C**) Mean changes in X and Y between 10s images of individual cells: parental (triangles); *cpnA^−cre^* (squares); *cpnA^−^* (circles); solid symbols are for cAMP gradient; open symbols are for buffer control. Mean changes in X and Y were analyzed by *t*-tests, * *p* < 0.05. (**D**) Mean velocities of individual cells within the Dunn chamber. (**E**) Mean circularity of cells within the Dunn chamber at a single timepoint. Black bars indicate cAMP gradient; Gray bars indicate buffer control; *n* = 94 for parental in buffer; *n* = 128 for parental in cAMP; *n* = 53 for *cpnA^−cre^* in buffer; *n* = 60 for *cpnA^−cre^* in cAMP gradient; *n* = 74 for *cpnA^−^* in buffer; *n* = 57 for *cpnA^−^* in cAMP gradient; *n* = 53 for *cpnA^−^* pulsed in buffer; *n* = 47 for *cpnA^−^* pulsed in cAMP gradient. Mean velocities and circularities were analyzed by a 2-way ANOVA with post-hoc comparisons with the Tukey method, *p* < 0.05. Means with different letters are significantly different.

To examine the cAMP chemotaxis defect at the individual cell level and examine cell behavior in a shallow gradient using 5 μM cAMP over a short period of time, we used time-lapse imaging to track individual cell movement. Cells were starved for six hours and then placed in a Dunn chamber within a cAMP gradient or a buffer control with no cAMP. Images were taken every 10 s for 10 min to create time-lapse movies. ImageJ was used to track the movement of all cells that appeared viable and stayed within the field of view during each movie. Figure 6A shows a representative set of cell trajectory data compiled from three different areas of the Dunn chamber within a single trial. 

Individual cells for each cell type were analyzed for directionality, velocity, and circularity from three different areas of the Dunn chamber in each of two separate trials (Figure 6C–E). To measure directionality, the changes in x and y coordinates every 10 s for 10 min were averaged for each cell. The mean of the average changes in x and y for all cells tracked is shown in Figure 6C. The parental cells within a cAMP gradient on average moved 0.71 ± 0.019 μm in the y direction (towards the cAMP gradient) and 0.071 μm ± 0.019 μm in the x direction in 10s, indicating directional movement in the direction of the cAMP gradient. On average, the parental cells with no gradient and the *cpnA* KO cells with or without a gradient exhibited very little average movement in either the y or x direction (Figure 6C). 

Parental cells within a cAMP gradient moved the fastest (9.8 ± 0.51µm/min). Parental cells with no gradient moved more slowly (6.2 ± 0.92 µm/min) than cells within a cAMP gradient. The *cpnA* KO cells moved 2.5–3× more slowly than parental cells within a cAMP gradient with mean velocities of 3.30 ± 0.42 µm/min for *cpnA^cre−^* cells and 4.02 ± 0.62 μm/min for *cpnA^−^* cells. *cpnA* KO cells showed no significant difference in velocity within a cAMP gradient or no gradient (Figure 6D). 

We also noticed that *cpnA* KO cells did not look as elongated as parental cells (Figure 6B). Therefore, we used ImageJ to measure the roundness of individual cells (Figure 6E). A value of 1.0 means a perfectly round shape, while the value approaches 0 as the cell shape is more elongated or polar. Parental cells within the cAMP gradient were the least round or most polar with a roundness factor of 0.38 ± 0.015. Parental cells in no gradient were less polar (0.48 ± 0.029) than cells within a gradient. *cpnA* KO cells were significantly rounder and less polar than parental cells and were similarly shaped in a gradient and no gradient with polarity ranging from 0.70–0.75. This data indicates that *cpnA* KO cells are defective in creating and maintaining a polar shape. We also pulsed *cpnA^−^* cells with cAMP every six hours prior to imaging to mimic normal cAMP signaling after starvation; pulsing cells did not change any of the chemotaxis parameters measured as compared to non-pulsed *cpnA^−^* cells. 

Together, these data indicate that *cpnA* KO cells exhibit defects in motility, polarity, and directional movement towards cAMP. However, the data from the chemotaxis assays on agar indicated that *cpnA* KO cells had a defect in directionality, but not motility. The different results from these two types of chemotaxis assays could be due to the differences in chemoattractant concentration, time period of the assay, and substrate surface. 

### 3.6. cpnA KO Cells Exhibit Reduced Actin Polymerization in Response to cAMP Stimulation

**Figure 7:**
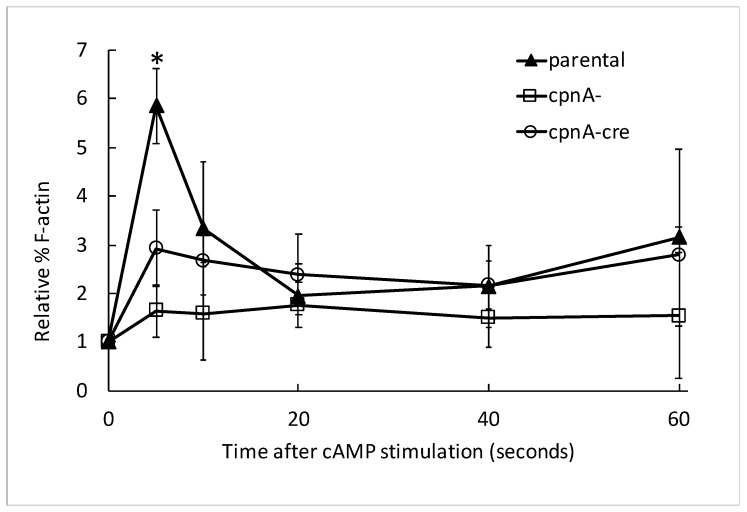
*cpnA* KO cells exhibit reduced actin polymerization in response to cAMP stimulation. Parental and *cpnA* KO cells (*cpnA^−^* and *cpnA^−cre^*) cells were pulsed with cAMP every 6 min for 5 h to induce development. Cells were stimulated with cAMP. At various timepoints after stimulation, cells were treated with Triton X-100, and the insoluble actin cytoskeleton was pelleted. Pellets and supernatants were analyzed by SDS-PAGE. The percent of actin in the pellet (F-actin) was determined using densitometry. The densitometry data were normalized to the 0 s timepoint, and three experiments from the same pulsed cell population were averaged. The means from two of *cpnA* KO or three parental pulsed cell populations were averaged. Error bars = standard error. The % of F-actin in the parental strain at 5 s was significantly different from the 0 s timepoint, and significantly different from the relative % F-actin in the *cpnA^−^* and *cpnA^−cre^* cells at the 5 s timepoint. (*t*-tests, * *p* < 0.05).

*Dictyostelium* cells respond to cAMP stimulation with a rapid increase of actin polymerization at the leading edge of cells undergoing chemotaxis, with actin polymerization peaking at ~5 s after stimulation [23,24,25,26]. We used a global cAMP stimulation assay to induce rapid actin polymerization at the plasma membrane. This assay allows us to distinguish defects in actin filament regulation from other defects that affect chemotaxis. Cells were pulsed with 50 nM cAMP every 6 min for 5 h in shaking suspension to mimic the developmental process. Cells were then stimulated with 100 μM cAMP, and cell samples were removed at 5, 10, 15, 20, 40, and 60 s timepoints after stimulation. Cell samples were immediately treated with 1% Triton X-100 and centrifuged to separate the insoluble actin cytoskeleton from soluble proteins. Proteins of the pellet, containing F-actin, and the supernatant, containing G-actin, were separated by SDS-PAGE and stained with Coomassie Blue. The actin bands on the gels were analyzed by densitometry using ImageJ. The parental strain responded to cAMP stimulation with a rapid increase in actin polymerization peaking at the 5-s timepoint (Figure 7). Both *cpnA* KO strains also exhibited a rapid increase in actin polymerization in response to cAMP stimulation, but the percentage of F-actin vs. G-actin was not as high as in the parental strain. (Figure 7). The % of F-actin in the parental strain at 5 s was significantly different from the 0 s timepoint (one-tailed *t*-test, *p* = 0.012), and significantly different from the *cpnA^−^* (one-tailed *t*-test, *p* = 0.01) and *cpnA^−cre^* (one-tailed *t*-test, *p* = 0.04) cells at the 5 s timepoint, suggesting that *cpnA* KO cells have a reduced actin polymerization response to cAMP. These results indicate that cells lacking *cpnA* are defective in their actin polymerization response to cAMP stimulation and suggest that their inability to move directionally in a cAMP gradient may be due to a defect in actin filament dynamics.

### 3.7. cpnA^−^ Cells Exhibit Increased Adhesion

**Figure 8:**
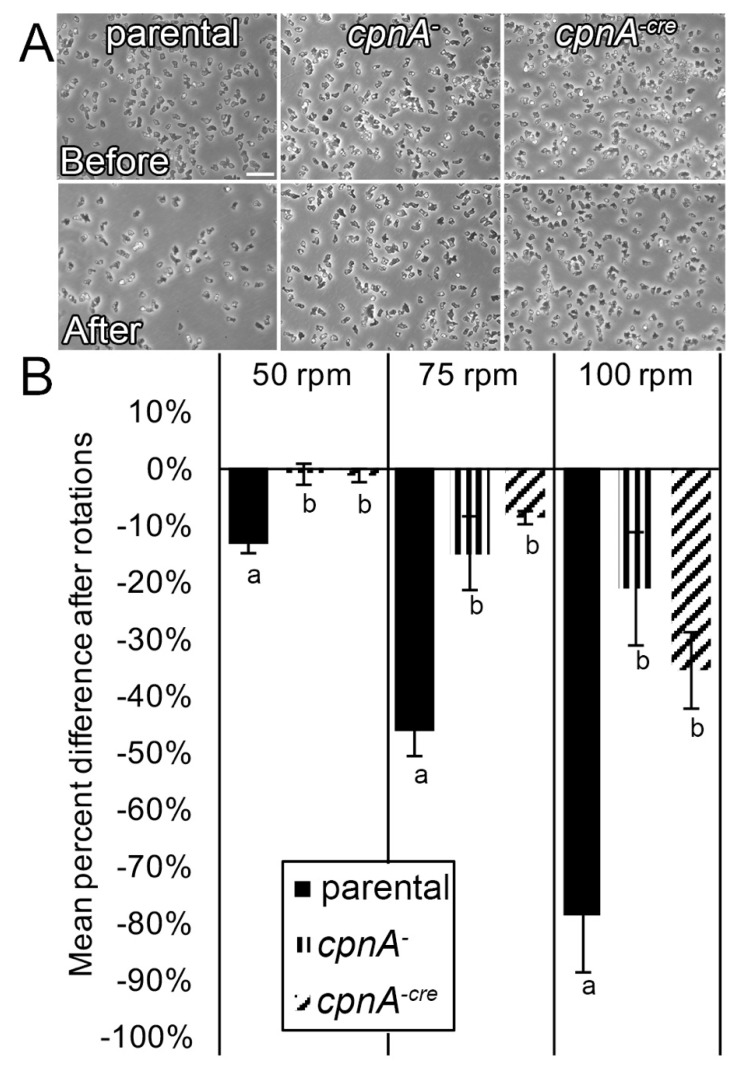
*cpnA* KO cells exhibit increased adhesion. Parental and *cpnA^−^* KO cells were plated on 35x10 mm Petri dishes and imaged with phase-contrast microscopy before and after rotation on a benchtop orbital shaker at 50, 75, and 100 RPM. The number of cells in each image were counted using the Cell Counter plugin in ImageJ and averaged for each RPM for three trials. (**A**) Images before and after rotation at 75 RPM. Scale bar represents 25 µm. (**B**) Percent difference of cells remaining adhered to the bottom of Petri dishes after rotation. Error bars represent standard error. Means for each rotation speed with different letters are significantly different (*t*-tests, *p* < 0.05).

Another cell property dependent on actin filaments is cell adhesion [27]. To test the adherent properties of *cpnA* KO cells to a surface, we plated cells on small Petri dishes and then placed them on a benchtop orbital shaker. The force created by the orbital shaking pulled cells off the bottom of the dishes and into the solution, and detached cells were removed from the plates. The number of cells present before and after shaking were counted in phase-contrast images acquired from five marked locations on the Petri dish (Figure 8A). Nearly twice as many *cpnA* KO cells remained adhered to the bottom of the Petri dishes compared to wildtype when cells were subjected to shaking at 50, 75, and 100 RPM (Figure 8B). Although this experiment was done with vegetative cells and not starved cells, the increased adhesion of *cpnA* KO cells may contribute to their decreased motility observed in the Dunn chamber chemotaxis assays. 

## 4. Discussion

In this study, actin was identified as a binding protein of CpnA using column chromatography and immunoprecipitation. In addition, purified GST-CpnA was shown to bind F-actin, suggesting that CpnA can bind directly to actin filaments. However, we cannot rule out the possibility that another protein that may have been co-purified with the GST-CpnA mediated this interaction. Some of the human copines have also been reported to bind to actin filaments [9]. In a yeast two-hybrid study, the A domains of human copines I, III, and IV were used as bait and β-actin was identified as a binding partner for all three. In addition, the interaction with β-actin was confirmed in an in vitro binding assay [9]. 

To investigate whether CpnA has a role in an actin-dependent process in vivo, we characterized the chemotaxis properties of *cpnA* KO cells. Chemotaxis to cAMP was assessed for *cpnA* KO cells in two different types of assays that differed with respect to cAMP concentration, substrate surface, and time period. The results of the chemotaxis assays on an agar substrate over a 3-hour period indicated that populations of *cpnA* KO cells were able to sense and move in response to a steep cAMP gradient, but were not able to move in the direction of the chemoattractant. This behavior may be indicative of oversensitivity to cAMP in that the *cpnA* KO cells are overwhelmed by the signal and are not able to sense the direction of the signal.

The results of the Dunn chamber assays where individual cells were imaged moving within a shallower cAMP gradient on glass over a 10-min period indicated that *cpnA* KO cells were defective in both motility and directional movement. Therefore, in contrast to the agar assays, *cpnA* KO cells not only had a defect in directional movement, but they also moved more slowly than parental cells. The decreased speed on glass could be attributed to the increased adherence of *cpnA* KO cells. Both the Dunn chamber and agar assays indicated that *cpnA* KO cells were impaired in directional movement towards cAMP. In addition to a defect in directional movement, *cpnA* KO cells within a cAMP gradient did not have the characteristic elongated, polar shape of a chemotactic cell, indicating a defect in cell polarity. 

A defect in directional movement or cell polarity could be caused by a multitude of defects that are not directly related to a specific role in regulating the actin cytoskeleton. Therefore, we tested whether *cpnA* KO cells have a normal actin polymerization response to global cAMP stimulation in the absence of chemotaxis. We found that the fast actin polymerization response is not as robust in *cpnA* KO cells as observed in the parental cells, suggesting that CpnA’s role in chemotaxis is related to the regulation of the actin cytoskeleton. 

In a recent study, we showed that five *Dictyostelium* copines, including CpnA, translocated from the cytoplasm to the plasma membrane and back to the cytoplasm in response to cAMP stimulation [15]. Compared to the other copines, the translocation of CpnA to the plasma membrane in response to cAMP was the most difficult to observe, in that the amount of protein found at the membrane was much lower and appeared more diffuse than the other copines. This diffuseness may be more indicative of an association with actin filaments than the plasma membrane [28]. The translocation response of CpnA to cAMP stimulation was also the fastest, with CpnA going to the membrane first and coming off the membrane first compared to the other copines [15]. The translocation timing of CpnA is similar to the fast actin polymerization response to cAMP stimulation [15,23,24,25,26], suggesting that CpnA may translocate to the plasma membrane or cell cortex to either activate or inhibit other actin regulating proteins or regulate actin filaments directly. 

This rapid translocation of CpnA to the plasma membrane in response to cAMP is similar to what has been observed for several other proteins functioning within chemotactic signaling pathways [29,30,31]. Studies using a tagged Ras-GTP binding domain peptide have shown that upon cAMP stimulation, activated Ras transiently localizes to the plasma membrane and this recruitment is not dependent on actin filaments [31]. Another protein that is rapidly recruited to the plasma membrane is PI3K; this recruitment is dependent on actin filaments [31]. As a calcium-dependent membrane-binding protein, CpnA may be responding to the rapid transient increase in intracellular calcium concentration that occurs after cAMP stimulation [32,33,34,35]. However, other copines (CpnC and CpnF) translocate to the plasma membrane in response to cAMP more slowly than CpnA [15] and this response is much later than the transient calcium concentration increase observed with cAMP stimulation, suggesting that the translocation of at least some copines to the membrane is not in direct response to a rise in calcium concentration. Furthermore, although cAMP stimulation triggers a transient rise in calcium, the role of calcium in chemotaxis in *Dictyostelium* is not clear [29,36]. Deletion of the IP3 receptor necessary for cAMP triggered calcium release does not affect chemotaxis, indicating the fast transient rise in calcium is not necessary for chemotaxis [36]. 

There are a few known examples of mammalian copines that translocate to the plasma membrane in response to a stimulus to regulate an actin-based process. Human copine 3 was shown to translocate from the cytosol to the plasma membrane in response to a rise in calcium concentration and colocalize with activated ErbB2 receptors in breast cancer cells. Copine 3 also localized to focal adhesions in migrating cells and was required for ErbB2-dependent cell migration [37]. Copine 6 was shown to translocate to the membranes of dendritic spines in response to synaptic activity in mouse hippocampal neurons. Copine 6 was also shown to interact with and activate Rac1, a Rho GTPase involved in the regulation of the actin cytoskeleton, to mediate changes in dendritic spine morphology important in learning and memory [8]. Neither of these studies indicated a direct interaction with actin filaments. Although our in vitro data suggests that CpnA interacts with actin filaments directly, CpnA, may function more like Copine 6, and indirectly regulate actin filaments by regulating other proteins, like Rac proteins. In *Dictyostelium*, several Rac proteins have been shown to be involved in chemotaxis by regulating actin polymerization [38,39]. 

Our previous studies on *cpnA^−^* cells are consistent with CpnA having a role in regulating the actin cytoskeleton [11,12,13]. *cpnA^−^* cells have defects in cytokinesis, contractile vacuole function, and development [11,12,13]. All of these processes depend on the actin cytoskeleton for proper function [40,41,42]. In this study, we showed that *cpnA* KO cells had defects in chemotaxis, polarity, and adhesion, which are all dependent on the actin cytoskeleton. Therefore, the assortment of defects observed in *cpnA* KO cells may be due to a defect in the regulation of actin filament dynamics. 

## Supplementary Materials

The following are available online at https://www.mdpi.com/2073-4409/8/7/758/s1, Figure S1: Verification of *cpnA^−^* KO mutants in *Dictyostelium*, Figure S2: Purification of GST-CpnA from *Dictyostelium* cells, Figure S3: Immunoprecipitated GFP-CpnA and GFP-Ado bind to F-actin, Figure S4: Verification of GFP, GFP-Ado, and GFP-CpnA. Table S1: Mass Spectrometry Data accompanying Figure 1. Table S2: Mass Spectrometry Data accompanying Figure 2.
