# Supplementary material for: Copine A Interacts with Actin Filaments and Plays a Role in Chemotaxis and Adhesion
Source: Cells. 2019 Jul 21;8(7):758. doi: 10.3390/cells8070758 (PMC6679068; doi:10.3390/cells8070758)
Supplement: Supplementary file 1 [file cells-08-00758-s001.pdf]

Copine A interacts with actin filaments and plays a role in chemotaxis and adhesion

Matthew J. Buccilli[1], April N. Ilacqua[1], Mingxi Han[1], Andrew A. Banas[1], Hanqian Mao[1], Samantha P. Perry[1], Tasha S. Salter[1], David Loiselle[2], Timothy A.J. Haystead[2], and Cynthia K. Damer[1*]

[1] Department of Biology, Central Michigan University, Mount Pleasant, MI 48859
bucci1mj@cmich.edu; ilacq1an@cmich.edu; mingxihan@gmail.com; banasandy@live.com; mao1hq@gmail.com; perry1sp@cmich.edu; smtasha@umich.edu

[2] Department of Pharmacology and Cancer, Duke University Medical Center, Durham, NC 27708
david.loiselle@duke.edu; timothy.haystead@duke.edu

Supplementary Files:

Figure S1. Verification of *cpnA*$^-$ KO mutants in *Dictyostelium*
Figure S2. Purification of GST-CpnA from *Dictyostelium* cells.
Figure S3. Immunoprecipitated GFP-CpnA and GFP-Ado bind to F-actin.
Figure 4S: Verification of GFP, GFP-Ado, and GFP-CpnA immunoprecipitation.
Table 1S. Mass Spectrometry Data accompanying Figure 1.
Table 2S. Mass Spectrometry Data accompanying Figure 2.

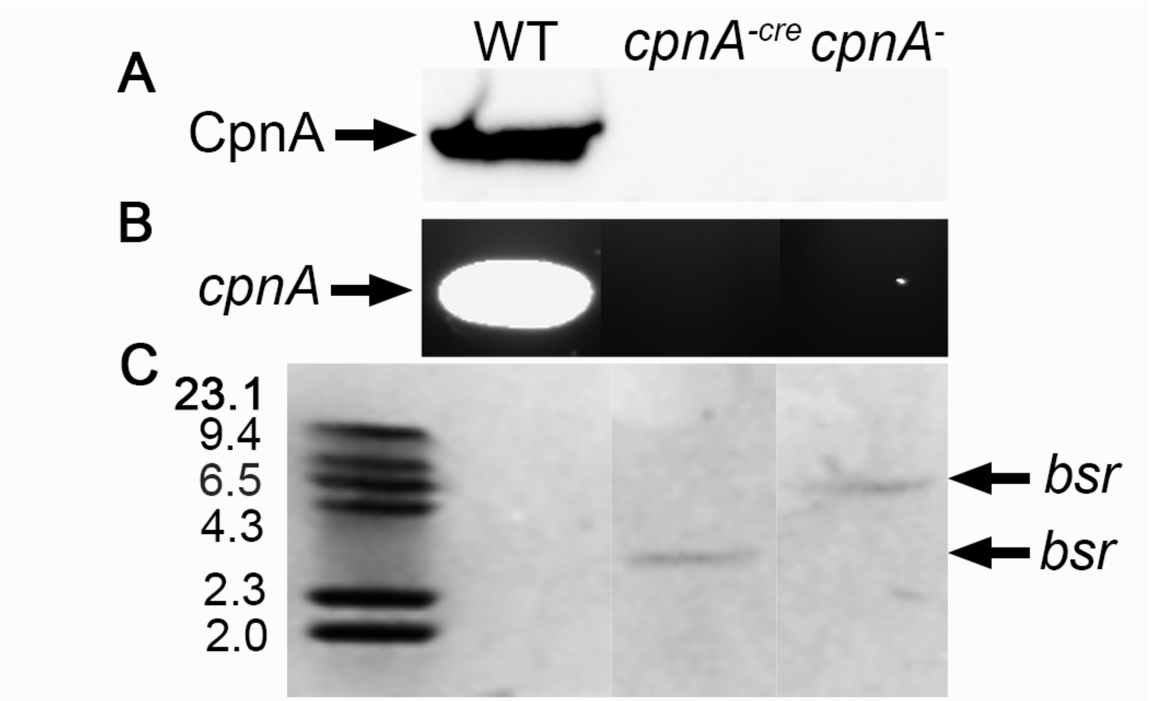

Figure 1S. Verification of *cpnA*$^{-}$ KO mutants in *Dictyostelium*. Wild-type (WT) and two different *cpnA* KO cells lines (*cpnA*$^{-}$ and *cpnA*$^{-cre}$) were analyzed by Western blot (A), PCR (B), and Southern blot (C). A. Whole cell samples were analyzed by Western blot with an antibody to CpnA. B. Cell lysates were used in PCR to amplify a small region in the middle of the *cpnA* gene. C. Genomic DNA was purified from cells and digested with *BglII*. The digested DNA was used in a Southern blot with a DNA probe made to the *bsr* gene. When the *cpnA* gene is replaced by the *bsr* gene, the *bsr* gene is found in a 7.12 kb *BglII* DNA fragment (*cpnA*$^{-}$). The *bsr* cassette used to make the *cpnA*$^{-cre}$ cells has an additional *BglII* site creating a 2.7 kb fragment containing *bsr*.

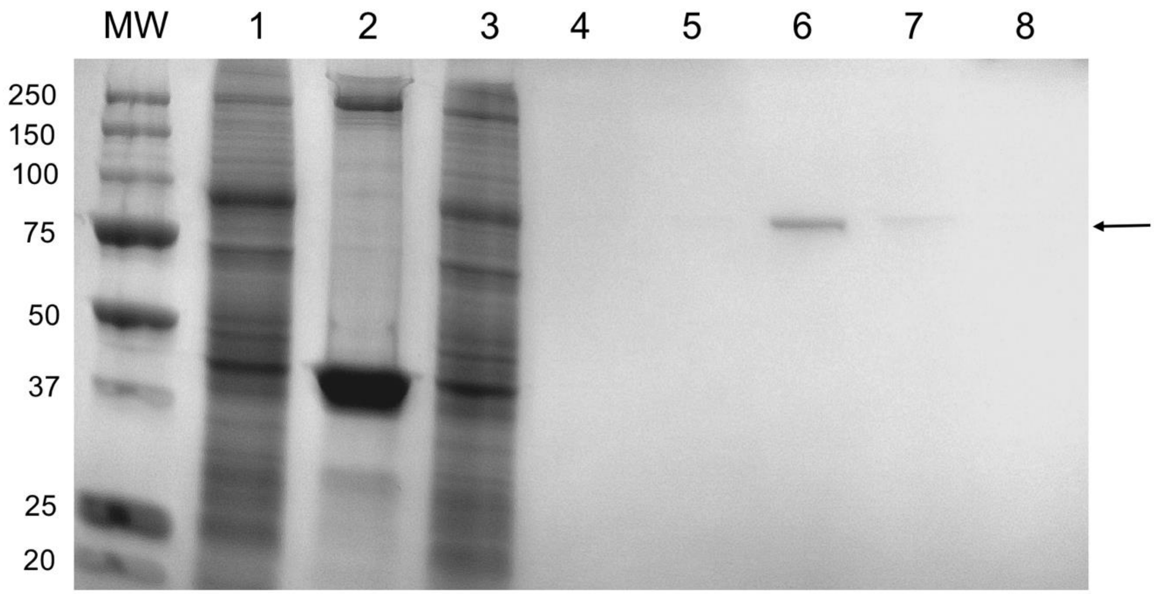

Figure 2S. Purification of GST-CpnA from *Dictyostelium* cells. A GST-tagged version of CpnA was expressed in wild-type *Dictyostelium* cells and the GST-CpnA was purified by glutathione agarose chromatography. First, cells were lysed and all membranes were pelleted. Samples from the supernatant (1) and the pellet (2) were run on the gel. The cell lysate was incubated with glutathione agarose beads. The beads were pelleted and washed three times (3, 4, 5). GST-CpnA was eluted with glutathione (6, 7, 8).

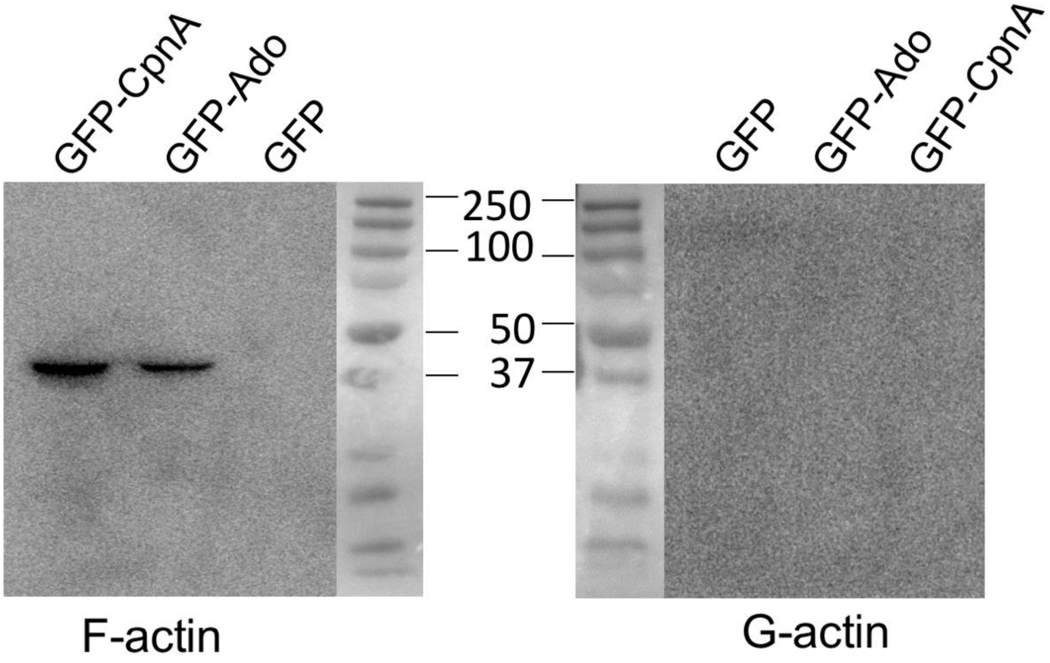

Figure 3S. Immunoprecipitated GFP-CpnA and GFP-Ado bind to F-actin. Immunoprecipitations with an antibody to GFP were performed with cells expressing GFP, GFP-Ado, and GFP-CpnA. Precipitated proteins were incubated with F-actin or G-actin and precipitated again. IPs were analyzed using a Western blot with an antibody to actin. Only sometimes did we see F-actin precipiate with GFP-Ado.

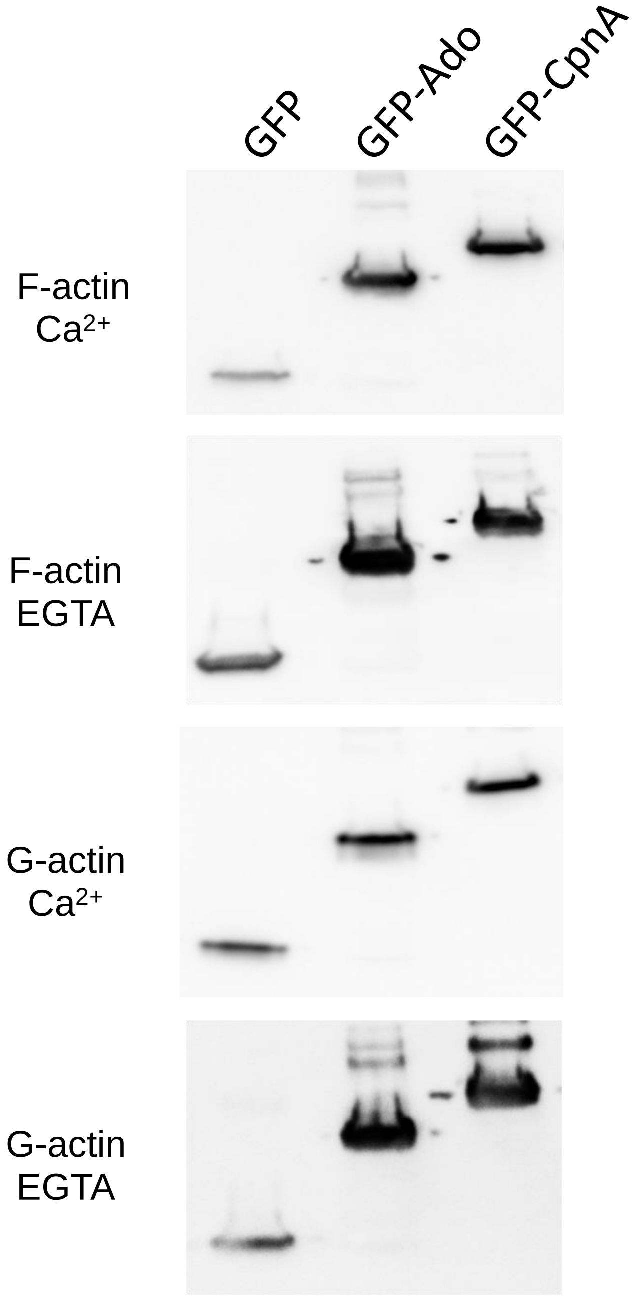

Figure 4S: Verification of GFP, GFP-Ado, and GFP-CpnA immunoprecipitation. Immunoprecipitations with an antibody to GFP were performed with cells expressing GFP, GFP-Ado, and GFP-CpnA with either calcium or EGTA in the buffer. Precipitated proteins were incubated with F-actin or G-actin and precipitated again. IPs were analyzed using a Western blot with an antibody to actin (Figure 4) and GFP to verify that GFP and GFP-fusion proteins were precipitated. GFP blots from the blots shown in Figure 4 are shown here. The amount of precipitated protein did vary among the different trials, but this did not correlate with whether actin was bound or not.
